# Abdominal Adiposity Correlates with Adenotonsillectomy Outcome in Obese Adolescents with Severe Obstructive Sleep Apnea

**DOI:** 10.1155/2012/351037

**Published:** 2012-11-18

**Authors:** Gustavo Nino, Maria J. Gutierrez, Anjani Ravindra, Cesar L. Nino, Carlos E. Rodriguez-Martinez

**Affiliations:** ^1^Penn State Sleep Research and Treatment Center, Pennsylvania State University College of Medicine, Hershey, PA, USA; ^2^Department of Pediatrics, Pennsylvania State University College of Medicine, Hershey, PA, USA; ^3^Division of Pediatric Pulmonary and Sleep Medicine, Penn State Hershey Children's Hospital, Pennsylvania State University College of Medicine, 500 University Drive, Hershey, PA 17033-0850, USA; ^4^Division of Allergy and Immunology, Pennsylvania State University College of Medicine, Hershey, PA, USA; ^5^Department of Electronics Engineering, Javeriana University, Bogota, Colombia; ^6^Department of Pediatrics, School of Medicine, Universidad Nacional de Colombia, Bogota, Colombia; ^7^Department of Pediatric Pulmonology and Pediatric Critical Care Medicine, School of Medicine, Universidad El Bosque, Bogota, Colombia; ^8^Research Unit, Military Hospital of Colombia, Bogota, Colombia

## Abstract

*Background*. Obese adolescents with Obstructive Sleep Apnea (OSA) have a unique pathophysiology that combines adenotonsillar hypertrophy and increased visceral fat distribution. We hypothesized that in this population waist circumference (WC), as a clinical marker of abdominal fat distribution, correlates with the likelihood of response to AT. *Methods*. We conducted a retrospective cohort study of obese adolescents (BMI ≥ 97th percentile) that underwent AT for therapy of severe OSA (*n* = 21). We contrasted WC and covariates in a group of subjects that had complete resolution of severe OSA after AT (*n* = 7) with those obtained in subjects with residual OSA after AT (*n* = 14). Multivariate linear and logistic models were built to control possible confounders. *Results*. WC correlated negatively with a positive AT response in young adolescents and the percentage of improvement in obstructive apnea-hypopnea index (OAHI) after AT (*P* ≤ 0.01). Extended multivariate analysis demonstrated that the link between WC and AT response was independent of demographic variables, OSA severity, clinical upper airway assessment, obesity severity (BMI), and neck circumference (NC). *Conclusion*. The results suggest that in obese adolescents, abdominal fat distribution determined by WC may be a useful clinical predictor for residual OSA after AT.

## 1. Introduction 

Obstructive Sleep Apnea (OSA) is characterized by recurrent episodes of partial or complete upper airway obstruction, resulting in oxygen desaturation and sleep disruption [1]. A number of risk factors likely influence airway patency during sleep, and thus the susceptibility for OSA. Adenotonsillar enlargement is the most commonly recognized anatomic cause for pediatric OSA [1], and obesity is the major risk factor during adulthood [2, 3]. As a result, adenotonsillectomy (AT) is considered the first line of therapy in most cases of pediatric OSA [4] but it is seldom effective in the adult population [5, 6], particularly in those who are obese [6, 7]. Obesity also increases the risk of residual OSA after AT in the pediatric population [8], however, the obesity features associated with decreased response to AT in children and adolescents are largely unknown. 

The anatomic and functional risk factors relating obesity to OSA are complex. Obesity leads to upper airway narrowing due to enlargement of soft palate, lateral pharyngeal walls, tongue, and parapharyngeal fat pads [9–13]. Along with these upper airway changes, obesity causes restrictive respiratory physiology primarily attributed to abdominal visceral fat accumulation [14]. The combination of narrow upper airway and lung restriction promotes a smaller and more collapse-prone upper airway that significantly increases OSA risk in all ages [15]. Indeed, in extremely obese adolescents (BMI ≥40 kg/m^2^), the reported prevalence of OSA is 55% [16]; in extremely obese adults the prevalence increases to 98% [17]. Interestingly, in the pediatric population visceral fat distribution is predictive of OSA severity independently of body mass index (BMI), which may explain why some obese children develop OSA and some do not [18].

In this context, there is emerging evidence suggesting that obese adolescents have a unique OSA pathophysiology. For instance, a recent study in young obese adolescents (mean age 12.5 ± 2.8 yr.) identified generalized overgrowth of adenoids and tonsils leading to upper airway restriction in subjects with OSA [19]. In the same study, there were no differences in soft palate, tongue, and mandible sizes in obese individuals with OSA compared to those without OSA. In contrast, obese adolescents with OSA had increased size of parapharyngeal fat pads and abdominal visceral fat, although the size of these tissues did not correlate with severity of OSA [19]. Collectively, these data suggested an important pathogenic role for adenotonsillar enlargement and body fat distribution in young obese adolescents with OSA. Our current work focused on how the clinical assessment of these factors may predict AT response in this population.

The primary goal of this study was to identify the clinical features that may predict response to AT in obese adolescents. Specifically, we hypothesized that in this population waist circumference (WC), as a clinical marker of abdominal fat distribution, correlates with the likelihood of response to AT independently of BMI. Secondary analysis evaluated the role of the clinical assessment of neck size, tonsillar size, and oropharyngeal narrowing (Mallampati score) predicting AT response in obese adolescents. Our hypotheses were tested using multivariate analysis contrasting WC and covariates in a group of subjects that had complete resolution of severe OSA after AT with those obtained in obese adolescents with severe OSA that did not resolve after AT. The resolution of OSA was clearly documented with clinical and polysomnographic objective evaluations before and after AT. The impact of the presented data is that it provides new information that may aid in the clinical assessment of obese adolescents with severe OSA, particularly in regard to likelihood of cure after AT. 

## 2. Methods

### 2.1. Subjects

We conducted a retrospective cohort study. Our study population included young adolescents (10–16 years old) selected from our database of all children that underwent routine overnight polysomnography (PSG) at the Penn State Sleep Research and Treatment Center between September 2009 and October 2011. Patients of both genders and all ethnicities were eligible for the study if: (1) they were obese (BMI ≥97th percentile for age and sex according to 2000 CDC growth charts for the United States), (2) had diagnosis of severe OSA defined as apnea–hypopnea index (OAHI) ≥10 events per hour on their initial PSG, (3) underwent AT for therapy of OSA, (4) had undergone a PSG within 6 months after AT, and (5) had documented clinical assessment of upper airway and fat distribution (neck, waist, and hip size) prior to AT. Patients were excluded if they had respiratory failure, central hypoventilation syndromes, congenital heart disease, severe developmental delay, cerebral palsy, craniofacial abnormalities, and neuromuscular disorders. Subjects were subdivided in those with “OSA resolution after AT” (*n* = 7) and those that did not have complete resolution of OSA after AT (*n* = 14). “OSA resolution after AT” was defined as the presence of all the following: (1) ≥90% reduction in obstructive apnea-hypopnea index (OAHI) after AT, (2) post-AT OAHI <5 events/hour, and (3) subjective improvement of OSA symptoms (i.e., snoring and excessive daytime sleepiness) in post-AT clinical visit. This study was approved by the Institutional Review Board of Penn State College of Medicine.

### 2.2. Sleep and Respiratory Recordings

#### 2.2.1. PSG Protocol

Standard pediatric overnight PSG was performed on all patients. For 9-10 hours, the patient's sleep was continuously recorded to a computerized system (Twin PSG software; Grass Technologies. Inc., West Warwick, RI, USA) and scored manually in 30-second epochs according to standardized criteria [20]. Polysomnography measurements included electroencephalograms (EEG) (C4-A1, O2-A1), right and left electrooculograms (EOG), electrocardiogram (ECG), mental-submental electromyogram (EMG), leg EMG, thoracic and abdominal wall motion (respiratory inductance plethysmography), pulse oximetry (with 2-s averaging time), end-tidal carbon dioxide monitoring (RespSense Capnograph, Grass Technologies. Inc., West Warwick, RI, USA), combined nasal/oral thermistor and nasal pressure (model TCT R, Grass-Telefactor, Inc.). Objective estimate of snoring during the PSG was obtained with a microphone attached to the neck (model 1250G Grass Technologies Inc., West Warwick, RI, USA). Body position and movements were determined by a sensor and confirmed by direct observation throughout the night along with routine infrared video monitoring. 

#### 2.2.2. PSG Scoring and Analysis

Sleep stages, arousal index, sleep efficiency, and respiratory events were scored according to standardized criteria [20]. Five sleep stages were identified (wake stage = W, stage 1 = N1, stage 2 = N2, stage 3 = N3, and stage REM = R). The OAHI included obstructive apneas, hypopneas, and mixed apneas. The minimum respiratory event duration was ≥2 respiratory cycles. Obstructive apneas were scored if there was an absence of airflow with continued respiratory effort. Obstructive hypopneas were scored if there was a discernible decrease in airflow of approximately 50% associated with either a ≥3% SaO2 desaturation and/or an arousal. Mixed apneas were scored if there was a discernible decrease in airflow with a period of no respiratory effort and a period of continued respiratory effort associated with either a ≥3% SaO2 desaturation and/or an arousal. 

### 2.3. Clinical Fat Distribution and Upper Airway Variables

#### 2.3.1. Obesity Anthropometric Measurements

Anthropometric data were obtained from electronic medical records (EMR) as they are performed routinely during PSG visit in our Sleep Research and Treatment Center (SRTC). Standing height and weight were obtained while patients were wearing lightweight clothing without shoes. Body mass index (BMI) was calculated using the formula weight (kg) divided by the square of height (m^2^). A nonelastic flexible tape measure was used to measure neck, waist, and hip circumference. Waist circumference (WC) was measured at the level of the umbilicus with the participants standing at the end of normal expiration. The hip circumference (HC) was measured at the greater trochanter. Neck circumference (NC) was measured horizontally at the level of the thyroid cartilage with head erect and eyes facing forward. Waist-to-hip ratio was calculated as WC divided by HC. 

#### 2.3.2. Oropharynx and Tonsillar Size Clinical Assessment

Upper airway variables were obtained from EMR data recorded during the clinical visit prior to PSG in Penn State Children's Hospital and Penn State SRTC. In our institution oropharynx and tonsillar size are routinely assessed using a standardized grading system. Oropharynx is evaluated using Mallampati's classification [1–4] with the tongue kept in place without the use of a tongue depressor, as previously described [21]. In grade 1, the tonsils, pillars, pharynx, and soft palate are clearly visible. In grade 2, the uvula and only the upper part of the pillars and tonsils are visible between the palate and the tongue. In grade 3, only the soft and hard palate are visible, while the tonsils, pillars, pharynx, and base of the uvula were hidden behind the tongue. In grade 4, only the hard palate is visible. Tonsillar size is also routinely evaluated using a grading system [1–4] as previously described [22]. In grade 1, the tonsils are hidden in the tonsillar fossa behind the anterior pillars. In grade 2, the tonsils are visible beyond the anterior pillars and occupy ≤50% of the pharyngeal space (the distance between the medial borders of the anterior pillars). In grade 3, the tonsils occupy between 50 and 75% of the pharyngeal space. In grade 4, the tonsils occupied ≥75% of the pharyngeal space (kissing tonsils).

### 2.4. Statistical Analysis

Data were analyzed using the software SAS version 9.2 or later (SAS Institute Inc., Cary, NC, USA). Means and proportions of main demographic and PSG variables (i.e., OAHI pre- and post-AT) were calculated for the entire study population, as well as stratified according to AT response status. The effect of the upper airway variables (Mallampati score and tonsillar size) and obesity parameters (BMI, WC, HC, NC and W/H ratio) were first described by summary statistics (mean/standard deviation) for individuals that responded to AT and those that did not respond to AT. For pair-wise relationships, two-sample *t*-test was used to compare the mean value of the continuous outcome measures; and chi-square test or Fisher's exact test were used to compare the proportion of positive signals for binary outcomes. Multivariate general linear models were built to study the joint effect of obesity parameters (predictors) in the percentage of OAHI improvement (linear regression models) or probability of resolution of OSA (logistic regression models) subsequent to AT surgery, with control of some possible confounders such as gender, age, BMI, and upper airway variables. Significance was taken at the *P* < 0.05 level.

## 3. Results

### 3.1. Study Population

Our study population (*n* = 21) was subdivided into one group that responded to AT (*n* = 7) and another group without OSA resolution after AT (*n* = 14). Response to AT was defined as obstructive apnea-hypopnea index (OAHI) reduction after AT of >90%, post-AT OAHI of <5 events/hour, and subjective improvement of OSA symptoms post-AT. Accordingly, one group included subjects with a dramatic OAHI mean reduction of 94% (pre-AT OAHI = 34.4/hour and post-AT OAHI = 1.32/hour) and the other group included subjects with a moderate OAHI mean improvement of 35% (pre-AT OAHI = 31.1/hour and post-AT OAHI = 19.7/hour). Comparison of demographic variables in these two groups revealed no significant differences (Table 1(a)). Baseline polysomnographic parameters such as obstructive apnea-hypopnea index (OAHI), arousal index, and oxygen desaturation nadir were comparable in both groups (Table 1(b)). 

### 3.2. Abdominal Adiposity Correlates Negatively with AT Response in Young Adolescents

**Figure 1:**
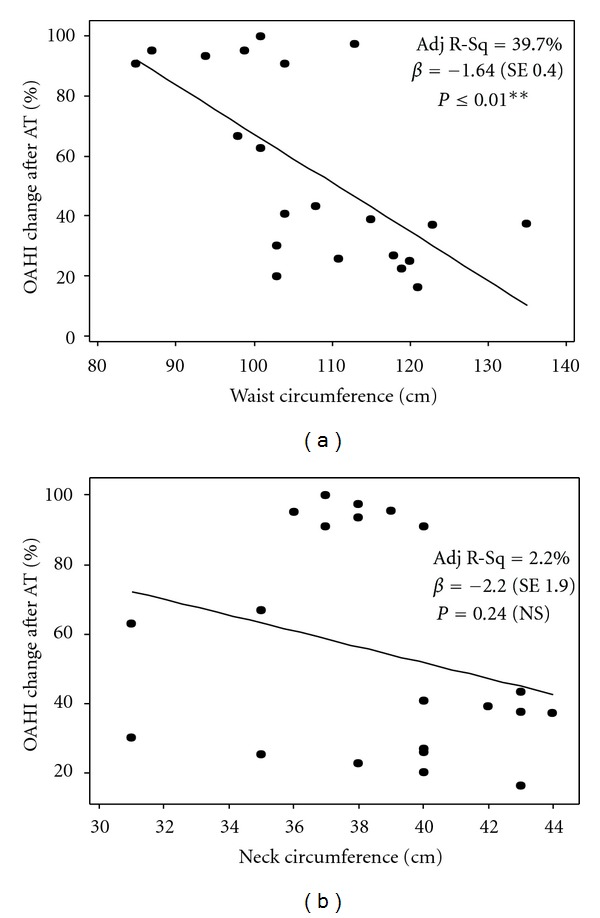
Univariate linear regression model (Fat distribution). Graphs demonstrate the correlation between either waist circumference (a) or neck circumference (b) and the percentage of apnea-hypopnea index (OAHI) change after adenotonsillectomy (AT) in obese adolescents. Adj R-Sq: adjusted coefficient of determination. *β*: parameter estimate. ± standard error (SE).

To investigate the role of clinical fat distribution in predicting AT response in young adolescents, we first compared neck, waist and hip circumferences in the group of obese adolescents with OSA resolution after AT with that seen in individuals without complete resolution of OSA after AT. As illustrated in Table 2(b), waist, and hip circumferences were significantly larger in the obese group that did not respond to AT (*P* ≤ 0.01) but neck size and waist-to-hip ratio were not significantly different. Univariate regression analysis revealed that waist circumference (WC) correlated negatively with the percentage of improvement in OAHI after AT (Adj *R* ^2^ = 39.7%, *β* = −1.64 ± SE  0.4, *P* ≤ 0.01) (Figure 1(a)) but neck circumference (NC) did not correlate significantly with AT response (Figure 1(b)). Extended analysis included multivariate predictive models built to assess the confounder effect of body mass index (BMI), gender, age, race, and neck size in the relationship between WC and the percentage of OAHI improvement (linear model) and the probability of OSA resolution (logistic model) following AT (Table 3). After adjusting for these covariables we found that the effect of WC in OSA resolution after AT is independent of BMI, gender, age, race, and neck size in young obese adolescents with severe OSA. The relationship between WC and AT response was not independent from waist-to-hip ratio as hip circumference (HC) also correlated negatively with OSA resolution after AT (data not shown). Collectively, these results suggest that clinical abdominal adiposity correlates negatively with AT response in young adolescents independently of demographic variables, obesity severity (BMI), and clinical neck fat distribution, as evaluated by NC. 

### 3.3. The Link between Abdominal Adiposity and AT Response Is Independent of Clinical Upper Airway Assessment

We next considered the role of clinical tonsillar size [1–4] and Mallampati scores [1–4] in predicting AT response in obese adolescents with severe OSA. Table 2(a) illustrates that there were no significant differences in the clinical grading of tonsillar hyperthrophy and oropharyngeal narrowing (Mallampati score) in the group with OSA resolution after AT relative to that seen in individuals without complete resolution of OSA after AT (Table 2(a)). Univariate analysis demonstrated that neither clinical tonsillar size nor Mallampati grading correlated with OSA outcome following AT (data not shown). Moreover, multivariate predictive models demonstrated that these upper airway variables did not modify the relationship between abdominal adiposity (WC) and AT response in obese adolescents with severe OSA (Table 3). 

### 3.4. OSA Severity (OAHI) Does Not Correlate with AT Outcome in Young Adolescents

**Figure 2:**
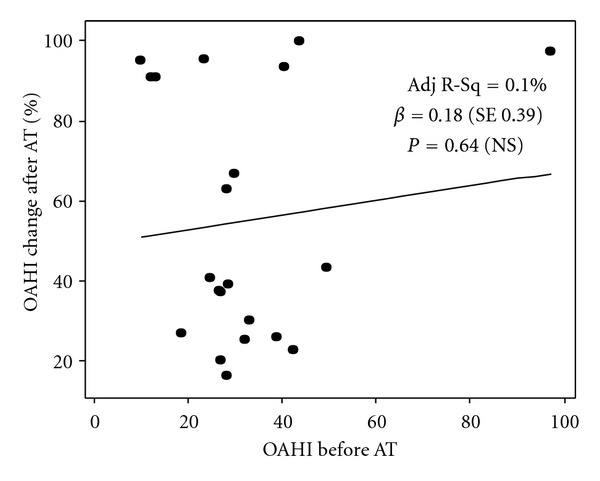
Univariate linear regression model (OSA severity). Graph demonstrates the correlation between obstructive apnea-hypopnea index (OAHI) before AT and the percentage of OAHI change after adenotonsillectomy (AT) in obese adolescents. Adj R-Sq: adjusted coefficient of determination. *β*: parameter estimate. ± standard error (SE).

To evaluate if OSA severity is a predictor of AT response in young adolescents, we compared OAHI in the group of obese adolescents with OSA resolution after AT with that seen in individuals without complete resolution of OSA after AT. Table 1(b) indicates that the PSG parameters indicative of OSA severity (i.e., OAHI) were comparable in both groups. Univariate regression analysis revealed that OAHI does not correlate with the percentage of improvement in OAHI after AT (Figure 2). Moreover, multivariate analysis demonstrated that OAHI and other PSG variables (i.e., oxygen desaturation nadir) did not modify the relationship between abdominal adiposity (WC) and AT outcome in obese adolescents with severe OSA (Table 3). 

## 4. Discussion

Prior investigations have focused on determining the effectiveness of adenotonsillectomy (AT) in the entire population of obese children and adolescents with Obstructive Sleep Apnea (OSA). These studies have demonstrated that AT improves OSA to some extent, but it does not resolve OSA in the majority of obese, pediatric patients [23]. Our study was designed to extend the knowledge of this relationship by focusing on the group of obese adolescents with OSA for whom AT seems to be a viable cure. Specifically, we aimed to identify the clinical features that may predict which obese adolescents with severe OSA may have a beneficial response to AT. For this purpose, we compared a subpopulation of obese adolescents who had complete resolution of severe OSA after AT with another group of obese adolescents who had residual OSA after undergoing AT. The main finding of this study was that the circumference of the subject's waist (WC) had a negative correlation with a positive response to AT in obese adolescents. Extended, multivariate analysis also identified that the effect of WC was independent of the severity of OSA, the severity of obesity (as measured by body mass index (BMI), clinical tonsillar size, oropharyngeal narrowing (Mallampati score), and the circumference of the neck (NC). Accordingly, these results suggested that markers of abdominal adiposity, that is, WC, may be useful clinical predictors for residual OSA after AT in this population. In addition, the lack of correlation of other clinical parameters with AT outcome highlights the need for novel, diagnostic tools to better identify obese adolescents who have the potential for being cured by AT. 

During this investigation, we evaluated three parameters in obese adolescents with severe OSA, that is, (1) *Clinical upper airway assessment: *consistent on pre-AT Mallampati score for oropharyngeal patency (grades 1–4) and standardized palatine tonsillar size (grades 1–4); (2)* Obesity parameters:* including BMI, neck circumference (NC), waist circumference (WC), hip circumference (HC), and waist-to-hip ratio; and (3)* OSA parameters in polysomnography (PSG)*: including obstructive apnea-hypopnea index (OAHI), the severity of snoring, oxygen nadir desaturation, and the degree of sleep fragmentation, expressed as arousal index (AI). We compared these parameters in obese adolescents who's AT treatment was successful with the same parameters in obese adolescents who had residual OSA after AT. As illustrated in Tables 1 and 2, no significant differences were observed in the clinical assessment of the airway, the severity of obesity (BMI), or PSG parameters between the two groups. Conversely, we found that abdominal adiposity, determined by WC, had a significant correlation with the patients' reduced response to AT. These findings are in general agreement with the prevailing notion that clinical adenotonsillar size does not correlate with the severity of OSA in obese children [24] and that BMI values alone do not seem to predict the severity of OSA in the pediatric population [25, 26]. In contrast, studies using magnetic resonance imaging (MRI) spectroscopy to quantify fat distribution in obese children with OSA [18] have identified a strong relationship between visceral adiposity and OSA, which is independent of BMI [18]. Interestingly, BMI does correlate with the severity of OSA in adults [27], which suggests that the pathogenic effects of obesity on OSA are different in children than in adults. 

In this context, it is noteworthy that NC, a parameter of neck fat distribution associated with the severity of OSA in adults [27, 28], was not correlated with AT outcome in our population of obese adolescents. The latter results may reflect maturational changes that modulate the upper airway tone and patency in children, adolescents, and adults [1, 29]. In this regard, prior investigations of the upper airway of young adolescents identified that the slope of the pressure-flow curve (SPF), which relates to the collapsibility of the upper airway [29], is significantly more flat in adolescents than it is in adults [29]. In other words, it is expected that the upper airway of young adolescents can tolerate the load of neck adiposity better than that of adults because the adolescents' upper airway is less likely to collapse. Conversely, our data supported the concept that abdominal adiposity is a critical obesity factor for OSA in adolescents and adults [30–32], and, thus, it may be a useful clinical parameter to predict residual OSA following AT, independently of age. 

Another important finding of this study was that obese adolescents had a heterogeneous response to AT. We identified that some obese teenagers with OSA can be cured completely by AT, regardless of their BMI, NC, oropharyngeal patency, or severity of OSA. For instance, an obese individual (BMI = 35 kg/m^2^) with very severe OSA (OAHI = 97/hour) had complete resolution of OSA after AT (97% decrease in OAHI) (outlier in Figure 2). This heterogeneity in the response of obese adolescents to AT suggests a unique OSA pathophysiology in this age group. Indeed, Arens et al. recently identified overgrowth of the upper airway lymphoid tissues in obese pediatric subjects with OSA [19]. In contrast to obese adults, this study revealed that the soft palate and tongue are not different in obese children with OSA relative to pediatric obese individuals without OSA [19]. The latter evidence, together with our current data describing the role of abdominal adiposity in obese adolescents with OSA, suggests that OSA in this population is a multifactorial condition that involves pediatric elements (overgrowth of upper airway lymphoid tissues) and adult OSA features (enhanced visceral adiposity distribution), resulting in a very heterogeneous phenotype that requires individualized assessment.

In summary, in this study, we investigated the clinical features that may predict response to AT in young obese adolescents with severe OSA. Although the sample size was small, our strength is that we limited this investigation to individuals with PSG-documented resolution of OSA. Our results demonstrated that: (1) abdominal adiposity, as measured by WC, is correlated negatively with AT response in young adolescents and (2) the effect of abdominal adiposity is independent of the severity of OSA, the severity of obesity (BMI), clinically-determined tonsillar size, oropharyngeal narrowing (Mallampati score), and the distribution of neck fat (NC). The latter information suggests that abdominal fat distribution plays a pivotal role in the pathogenesis of OSA in obese adolescents, and, thus, it may be a useful clinical parameter for predicting residual OSA after AT. Our data also illustrated the unique phenotypical variability of OSA in obese adolescents and highlighted the need for novel, diagnostic approaches for this population. Emergent techniques, such as three-dimensional MRI spectroscopy, can delineate upper airway structures and body-fat composition [9–13, 18, 19], and, thus, they could be used to investigate the specific phenotypical features of OSA in obese adolescents, including their responsiveness to AT. 

## Figures and Tables

**Table 1:** Demographic and polysomnographic profile of subjects. For quantitative variables, data are presented as mean ± standard error (SE). AT: adenotonsillectomy; OAHI: obstructive apnea-hypopnea index. For categorical variables, data are presented as count number (column percentage). *P* values are obtained by either two-sample *t*-test or chi-square test, depending on the type of variables.

Factors/variables	Response to AT (*n* = 7)	No response to AT (*n* = 14)	*P* value
(A) Demographic variables			
Gender			
Female	2 (28.6%)	3 (21.5%)	0.725
Male	5 (71.4%)	11 (78.5%)	0.725
Age (years): mean (SE)	12.1 (0.4)	12.7 (0.6)	0.478
Ethnicity			
White	4 (57.1%)	8 (57.1%)	1.0
Others	3 (42.9%)	6 (42.9%)	1.0

(B) Sleep study parameters			
OSA severity			
Pre-AT OAHI: mean (SE)	34.4 (11.6)	31.1 (2)	0.789
Post-AT OAHI: mean (SE)	1.32 (0.3)	19.7 (1.8)	<0.01**
% OAHI improvement after AT	94.6 (1.3)	35.2 (4.0)	<0.01**
Sleep efficiency (%): mean (SE)	80.3 (4.6)	83.3 (2.5)	0.580
Arousal index (events per hr): mean(SE)	15.7 (3.5)	13.6 (2.4)	0.627
SaO2 nadir (%): mean (SE)	84 (3.0)	83.9 (1.4)	0.983

(^∗∗^ *P* ≤ 0.01).

**Table 2:** Upper airway and obesity variables. For quantitative variables, data are presented as mean ± standard error (SE). BMI: body mass index. *P* values are obtained by two-sample *t*-test.

Factors/variables	Response to AT (*n* = 7)	No response to AT (*n* = 14)	*P* value
(A) Upper airway variables			
Tonsilar size 1–4: mean (SE)	2.43 (0.2)	2.36 (0.1)	0.773
Mallampati score 1–4: mean (SE)	2.71 (0.1)	2.86 (0.2)	0.551
(B) Obesity parameters			
BMI (kg/m^2^): mean(SE)	33.5 (2.1)	37.6 (1.5)	0.131
Neck size (cm): mean (SE)	37.8 (0.51)	38.2 (1.2)	0.411
Waist size (cm): mean (SE)	97.6 (3.7)	112.8 (2.8)	<0.01**
Hip size (cm): mean (SE)	100.1 (2.7)	116.2 (3.3)	<0.01**
Waist-hip ratio: mean (SE)	0.97 (0.03)	0.97 (0.01)	0.972

(^∗∗^ *P* ≤ 0.01).

**Table 3:** Multivariate regression analysis. Either regular linear regression or binary logistic regression was performed, depending on the type of outcome variable in the statistical model. For logistic regression, the odds ratios (OR) and their *P* values are reported; while for regular linear regression, the parameter estimates and their *P* values are reported.

	Response to AT (Y/N)	% of AHI change after AT
Waist size predictive model variables adjusted	OR, *P* value	Parameter estimate, *P* value
	(by multivariate logistic regression)	(by multiple linear regression)
(A) Demographic		
Waist circumference	**1.23,** ***P≤* 0.0**5*	**−1.92,** ***P* ≤ 0.0**1**
Age	0.78, *P* = 0.54	2.18, *P* = 0.49
Gender		
Female^#^		
Male	1.34, *P* = 0.42	−3.3, *P* = 0.82
Ethnicity		
White^#^		
Other	1.33, *P* = 0.33	−17, *P* = 0.23

(B) Obesity parameters		
Waist circumference	**1.25**, *** P≤* 0.0**5*	−**1.35**, *** P≤* 0.0**5*
BMI	0.94, *P* = 0.68	−1.32, *P* = 0.34
Neck size	0.89, *P* = 0.60	0.63, *P* = 0.86

(C) Upper airway variables		
Waist circumference	**1.18,** *** P≤* 0.0**5*	**−1.65,** ***P* ≤ 0.0**1**
Mallampati score	1.43, *P* = 0.8	−16.8, *P* = 0.12
Tonsilar size	0.55, *P* = 0.64	4.1, *P* = 0.7

(D) OSA variables		
Waist circumference	**1.33,** ***P≤* 0.0**5*	**−1.96,** ***P* ≤ 0.0**1**
Obstructive AHI	0.96, *P* = 0.29	0.30, *P* = 0.34
SaO2 nocturnal nadir	1.04, *P* = 0.78	9.2, *P* = 0.18
Snoring severity	0.29, *P* = 0.34	−0.53, *P* = 0.61

Note: **#**reference level.

(  ^∗∗^ *P* ≤ 0.01,^∗^ *P* ≤ 0.05 ).
